# Effect of an integral quality monitor on 4‐, 6‐, 10‐MV, and 6‐MV flattening filter‐free photon beams

**DOI:** 10.1002/acm2.13106

**Published:** 2020-12-03

**Authors:** Trang Hong Thi Nguyen, Haruna Yokoyama, Hironori Kojima, Naoki Isomura, Akihiro Takemura, Shinichi Ueda, Kimiya Noto

**Affiliations:** ^1^ Division of Health Sciences Graduate School of Medical Sciences Kanazawa University Kanazawa Japan; ^2^ Department of Radiological Technology Kanazawa University Hospital Kanazawa Japan; ^3^ Faculty of Health Sciences Institute of Medical, Pharmaceutical and Health Sciences Kanazawa University Kanazawa Japan

**Keywords:** beam quality, integral quality monitor (IQM), photon energy

## Abstract

**Purpose:**

To investigate the effect of an integral quality monitor (IQM; iRT Systems GmbH, Koblenz, Germany) on 4, 6, 10, and 6‐MV flattening filter‐free (FFF) photon beams.

**Methods:**

We assessed surface dose, PDD_20,10_, TPR_20,10_, PDD curves, inline and crossline profiles, transmission factor, and output factor with and without the IQM. PDD, transmission factor, and output factor were measured for square fields of 3, 5, 10, 15, 20, 25, and 30 cm and profiles were performed for square fields of 3, 5, 10, 20, and 30 cm at 5‐, 10‐, and 30‐cm depth.

**Results:**

The differences in surface dose of all energies for square fields of 3, 5, 10, 15, 20, and 25 cm were within 3.7% whereas for a square field of 30 cm, they were 4.6%, 6.8%, 6.7%, and 8.7% for 4‐MV, 6‐MV, 6‐MV‐FFF, and 10‐MV, respectively. Differences in PDD_20,10_, TPR_20,10_, PDD, profiles, and output factors were within ±1%. Local and global gamma values (2%/2 mm) were below 1 for PDD beyond *d*_max_ and inline/crossline profiles in the central beam region, respectively. The gamma passing rates (10% threshold) for PDD curves and profiles were above 95% at 2%/2 mm. The transmission factors for 4‐MV, 6‐MV, 6‐MV‐FFF, and 10‐MV for field sizes from 3 × 3 to 30 × 30 cm^2^ were 0.926–0.933, 0.937–0.941, 0.937–0.939, and 0.949–0.953, respectively.

**Conclusions:**

The influence of the IQM on the beam quality (in particular 4‐MV X‐ray has not verified before) was tested and introduced a slight beam perturbation at the surface and build‐up region and the edge of the crossline/inline profiles. To use IQM in pre‐ and intra‐treatment quality assurance, a tray factor should be put into treatment planning systems for the dose calculation for the 4‐, 6‐, 10‐, and 6‐MV flattening filter‐free photon beams to compensate the beam attenuation of the IQM detector.

## 1 INTRODUCTION

Quality assurance (QA) plays an important role in minimizing and preventing errors in radiation therapy. Dosimetric evaluation for treatment plans has routinely used ionization chambers, thermoluminescent dosimeters, optically stimulated luminescent dosimeters, and films as conventional QA techniques.^1^, ^2^, ^3^, ^4^ However, the disadvantage of these QA devices is that they have only been used for offline verification and cannot predict any unexpected errors in vivo dosimetry. Moreover, the gamma passing rates were reported to be insufficient for prediction of dose errors in QA of intensity‐modulated radiation therapy.^5^, ^6^ Beam output should be verified during treatment to avoid potential treatment errors such as equipment failure and wrong plan selection.^7^, ^8^ Therefore, some advanced *in vivo* QA methods were introduced to increase the dosimetric accuracy in vivo dosimetry such as point dosimeters, electronic portal imaging device dosimetry (EPID), transmission detectors, linac log file analysis, and dose accumulation methods.^9^, ^10^, ^11^, ^12^, ^13^, ^14^, ^15^, ^16^, ^17^, ^18^, ^19^ The point dosimeters and EPID are sensitive to linac and patient errors.^20^ Transmission detectors monitor linac performance with high sensitivity in real time.^20^ Linac log file analysis is sensitive to plan corruption errors.^20^ Dose accumulation methods are used to evaluate intrafraction movements and the multileaf collimator (MLC) tracking systems.^20^

A type of new transmission detector called an integral quality monitor (IQM) has been commercialized by iRT Systems GmbH, Koblenz, Germany. The IQM is mounted on a linear accelerator (linac) gantry head and uses an independent system monitor to provide on‐line beam monitoring that can detect treatment delivery errors that exceed an acceptance level.^9^ The IQM is a wedge‐shaped ionization chamber with a continuous spatial resolution and the large sensitive volume that can detect a small range of systematic MLC error compared with 0.6‐cc Farmer chamber (PTW 30013, Freiburg, Germany), Delta4 (ScandiDos, Uppsala, Sweden), and 2D‐array seven29 (PTW, Freiburg, Germany).^21^ However, when the IQM is placed in the beam path, it was reported that the IQM affected to be small yet statistically significant photon beam properties with the increase in the surface dose and beam attenuation of 6‐, 10‐, 15‐, 18‐MV, and 6‐, 10 ‐MV flattening filter‐free (FFF) X‐ray beams.^22^ With respect to the effect of IQM on beam quality, Casar et al.^22^ evaluated the surface dose, difference in the ratio of percentage depth dose at depths of 20 and 10 cm (PDD_20,10_), and transmission factors for field sizes from 1 × 1 to 20 × 20 cm^2^ for 6‐, 10‐, 15‐, and 18‐MV and for two FFF photon beams (6‐ and 10‐MV FFF). Islam et al.^9^ evaluated the surface dose, the profiles at 1.5 and 10 cm depths for 30 × 30 cm^2^ field and percent depth dose for 10 × 10 and 30 × 30 cm^2^ fields, and transmission factor for a field size of 10 × 10 cm^2^ for 6‐ and 18‐MV X‐ray beams. Hoffman et al.^23^ evaluated the surface dose, the profiles at 10 cm depth for 30 × 30 cm^2^ field and percent depth dose for 10 × 10 cm^2^ fields, and transmission factor for field sizes of 1 × 1 and 10 × 10 cm^2^ for 6‐, 10‐ and 15‐MV beams. As a novelty of this manuscript, we furnish a broad and complete view of the beam perturbation induced by IQM by evaluating the differences in the surface and build‐up region dose, PDD_20,10_, tissue phantom ratio at depths of 20 and 10 cm (TPR_20,10_), percentage depth dose (PDD) curves, inline and crossline profiles, transmission factor, and output factor measured with and without the IQM for field sizes of 3 × 3, 5 × 5, 10 × 10, 15 × 15, 20 × 20, 25 × 25, and 30 × 30 cm^2^ for 4‐, 6‐, 10‐, and 6‐MV flattening filter‐free (FFF) X‐ray beams.

The previous studies evaluated the influence of the IQM on photon energies of Elekta linacs (Synergy, Precise, and Versa HD (Elekta AB, Stockholm, Sweden)) with 6‐MV low‐energy photons, 10‐MV mid‐energy photons, and 15‐ and 18‐MV high‐energy photons.^9^, ^22^, ^23^ However, in this study, we used an Elekta Infinity linac (Elekta AB, Stockholm, Sweden) with 4‐MV low‐energy photons, 6‐MV mid‐energy photons, and 10‐MV high‐energy photons. In Japan, there is basically a difference in the number of FFF beams between Elekta Infinity and Elekta Versa HD. The Elekta Versa HD has two FFF beams with 10‐ and 6‐MV FFF X‐ray beams whereas the Elekta Infinity only selects one of the FFF beams. Outside Japan, the specifications of linac may differ slightly. The Elekta Infinity with Agility MLC (160 leaves with 1 cm leaf width) can be installed depending on the country. In Japan, the 4‐MV X‐ray beam is used as low‐energy photon instead of 6‐MV X‐ray beam because the 4‐MV X‐ray beam is routinely used for breasts with small configuration in Japan whereas the 6‐MV X‐ray beam or higher energy photon is used for large‐size breasts in US and Europe.^24^ Therefore, the beam shaping inside Elekta linac in Japan is different from global Elekta linac. The 4‐MV beam of Japan and 6‐MV beam of global Elekta were produced with the combination of the primary collimator in the open position and secondary filter at the position of the low‐energy X‐ray filter, as shown in Table 1. The 6‐MV beam of Japan and 10‐MV beam of global Elekta were delivered at the open position of the primary collimator and secondary filter at the mid‐energy X‐ray filter. The 10‐MV beam of Japan and 15‐MV beam of global Elekta were produced with the combination of the primary collimator at the filter position and secondary filter at the mid‐energy X‐ray filter. Because the configuration of Elekta linacs depends upon the situation of each country, the evaluation of effect of the linac variation for photon beam is necessary and the results obtained for the 6‐ and 10‐MV beams in this study should be comparable with published papers.

**Table 1:** Difference of photon delivery system between the Elekta Linac in Japan and the global Elekta Linac for low‐, mid‐, and high‐energy photons.

Energy Variations	Low‐energy	Mid‐energy	High‐energy
Primary collimator	Open position	Open position	Filter position
Secondary filter	Low‐energy X‐ray filter	Mid‐energy X‐ray filter	Mid‐energy X‐ray filter
Global	6 MV	10 MV	15 MV
Japan	4 MV	6 MV	10 MV

## 2 MATERIALS AND METHODS

### 2.A Measurements of PDD curves and inline/crossline profiles

The IQM detector is attached to a gantry head of the Elekta Infinity linac. All measurements were carried out on the Elekta Infinity with an Agility MLC system (160 leaves with 0.5 cm leaf width) (Elekta AB, Stockholm, Sweden). PDD with and without the IQM was measured from a depth of 30 cm up to the surface water in a cylindrical 3D Scanner v.3.3.1 water phantom (Sun Nuclear, Melbourne, FL) using a 0.125‐cm^3^ SNC125c chamber (Sun Nuclear, Melbourne, FL) and SNC Dosimetry™ scanning software v.3.4.0.26814 (Sun Nuclear, Melbourne, FL) at a source‐to‐surface distance (SSD) of 100 cm and field sizes of 3 × 3, 5 × 5, 10 × 10, 15 × 15, 20 × 20, 25 × 25, and 30 × 30 cm^2^. The field sizes were defined by a pair of sculpted diaphragms mounted orthogonally to MLC. The measurement of the inline and crossline profiles with and without the IQM was performed for field sizes of 3 × 3, 5 × 5, 10 × 10, 20 × 20, and 30 × 30 cm^2^ at depths of 5, 10, and 30 cm.

### 2.B Measurements of TPR_20,10_, transmission factor, and output factor

TPR_20,10_ was measured in a QWP‐07 water phantom (Quolita, Nagano, Japan) using a 0.6 cm^3^ TN30013 Farmer‐type ionization chamber (PTW Freiburg GmbH, Freiburg, Germany) at a source–chamber distance (SCD) of 100 cm for a field size of 10 × 10 cm^2^ with and without the IQM. TPR_20,10_ was obtained from the ratio of the absorbed doses at depths of 20 and 10 cm. The transmission factor and output factor were measured for all energies and field sizes of 3 × 3, 5 × 5, 10 × 10, 15 × 15, 20 × 20, 25 × 25, and 30 × 30 cm^2^ with and without the IQM. The field sizes of 5 × 5, 10 × 10, 15 × 15, 20 × 20, 25 × 25, and 30 × 30 cm^2^ were measured using the 0.6 cm^3^ TN30013 Farmer‐type ionization chamber (PTW Freiburg GmbH, Freiburg, Germany) whereas the field size of 3 × 3 cm^2^ was measured using a 0.13 cm^3^ CC13 compact ionization chamber (IBA Dosimetry GmbH, Schwarzenbruck, Germany). The transmission factor for a field size was defined as the ratio of the ionization charge with the IQM to that without the IQM at a reference depth of 10 cm in the water phantom.^25^, ^26^ The output factor for a certain field size with and without the IQM was defined as the ratio of the measured dose for an actual field size in the water phantom and that for a reference field of 10 × 10 cm^2^ at a depth of 10 cm.^25^ The differences of output factor with and without the IQM were calculated for all energies and field sizes.

### 2.C Evaluation of the effect of IQM on beam quality

The influence of the IQM on the surface and build‐up region dose was evaluated by the dose difference from the surface (*depth* = 0 cm) to depth of dose maximum (*d*_max_) of the PDD curves with and without the IQM.^27^ The PDD_20,10_, TPR_20,10_, PDD curves, and inline/crossline profiles, transmission factor, and output factor with and without the IQM were used to assess the effect of the IQM on beam quality of 4‐, 6‐, 6‐MV FFF, and 10‐MV X‐ray beams beyond *d*_max_. The difference between PDD curves and inline/crossline profiles measured with and without IQM should be within ±1%. The differences at the build‐up region dose, PDD_20,10_, TPR_20,10_, PDD curves and inline/crossline profiles, and output factor measured with and without the IQM were defined as follows:

(1) $$Difference\left(\%\right)=\frac{X_{with\;IQM}‐X_{without\;IQM}}{X_{without\;IQM}}\times 100$$

where $X_{with\;IQM}$ and $X_{without\;IQM}$ are the build‐up region dose, PDD_20,10_, TPR_20,10_, PDD curves, crossline and inline profiles, and output factor measured with and without IQM, respectively.

The PDD curves and crossline and inline profiles measured with IQM were compared with the corresponding PDD curves and crossline and inline profiles measured without IQM using a gamma function described by Low et al.^28^ We used dose difference (2%) and distance to agreement acceptance criteria (2 mm) for the gamma calculations. The gamma criteria (10% threshold and gamma passing rate above 95% at 2%/2 mm) was used. Local and global dose differences were analyzed for PDD curves and crossline/inline profiles, respectively.

## 3 RESULTS

### 3.A The effect of IQM on the surface and build‐up region dose

**Fig 1:**
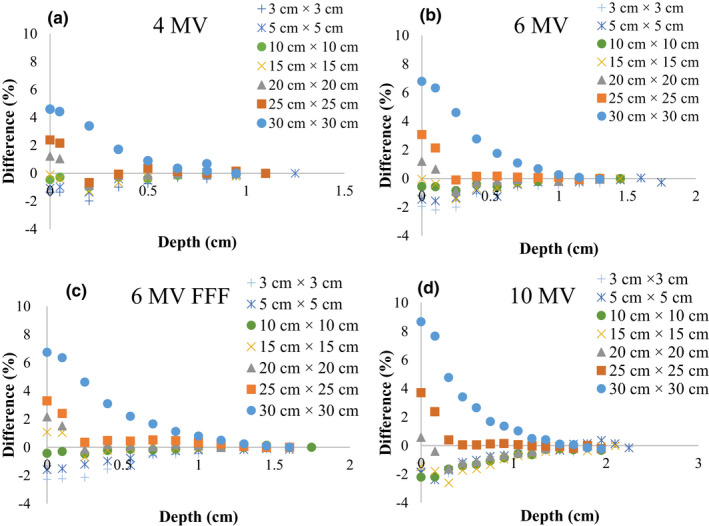
Dose differences from the surface to *d*_max_ of (a) 4‐MV, (b) 6‐MV, (c) 6‐MV FFF, and (d) 10‐MV X‐ray beams with and without the IQM for field sizes of 3 × 3, 5 × 5, 10 × 10, 15 × 15, 20 × 20, 25 × 25, and 30 × 30 cm^2^ and SSD of 100 cm.

Figures 1(a) to 1(d) show the dose differences from the surface to *d*_max_ of 4‐, 6‐, 6‐MV FFF, and 10‐MV X‐ray beams for field sizes of 3 × 3, 5 × 5, 10 × 10, 15 × 15, 20 × 20, 25 × 25, and 30 × 30 cm^2^ with and without the IQM. The dose differences at build‐up region of each energy for each field sizes from 3 × 3 to 30 × 30 cm^2^ decreased with increasing depth from the surface to *d*_max_ and fell to zero at *d*_max_. The differences in the surface dose of all energies for field sizes of 3 × 3, 5 × 5, 10 × 10, 15 × 15, 20 × 20, 25 × 25, and 30 × 30 cm^2^ with and without the IQM ranged from −1.4% (4‐MV) to −2.3% (6‐MV FFF), −1.1% (4‐MV) to −1.9% (10‐MV), −0.4% (6‐MV FFF) to −2.2% (10‐MV), −0.1% (6‐MV) to −1.5% (10‐MV), 0.6% (10‐MV) to 2.2% (6‐MV FFF), 2.4% (4‐MV) to 3.7% (10‐MV), and 4.6% (4‐MV) to 8.7% (10‐MV), respectively. The dose difference at the surface with a field size of 30 × 30 cm^2^ for the 10‐MV X‐ray beam was higher than that of the 4‐MV, 6‐MV, and 6‐MV FFF X‐ray beams (8.7%, 4.6%, 6.8%, and 6.7%, respectively).

### 3.B The effect of IQM on PDD_20,10_, TPR_20,10_, and PDD curves

**Fig 2:**
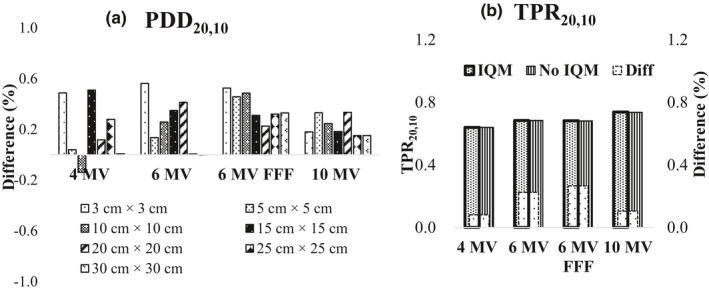
(a) Differences of PDD_20,10_ for field sizes of 3 × 3, 5 × 5, 10 × 10, 15 × 15, 20 × 20, 25 × 25, and 30 × 30 cm^2^ at an SSD of 100 cm and (b) comparison of TPR_20,10_ for field size of 10 × 10 cm^2^ at a depth of 10 cm and SCD of 100 cm measured with and without the IQM for 4‐MV, 6‐MV, 6‐MV FFF, and 10‐MV X‐ray beams.

**Fig 3:**
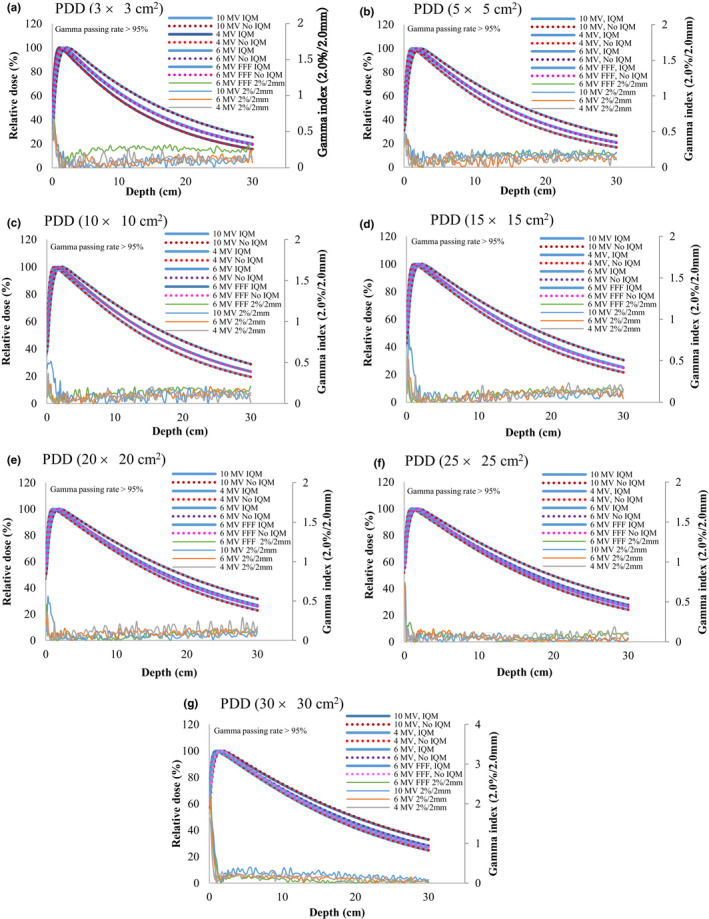
PDD curves and gamma values (2%/2 mm) for PDD of 4‐MV, 6‐MV, 6‐MV FFF, and 10‐MV X‐ray beams with and without the IQM for field sizes of (a) 3 × 3, (b) 5 × 5, (c) 10 × 10, (d) 15 × 15, (e) 20 × 20, (f) 25 × 25, and (g) 30 × 30 cm^2^ and an SSD of 100 cm.

The differences in PDD_20,10_ for all energies and all field sizes with and without the IQM were less than 0.6% [Fig. 2(a)]. The TPR_20,10_ values determined for all energies for a field size of 10 × 10 cm^2^ with the IQM agreed with the corresponding TPR_20,10_ data without the IQM within 0.3% [Fig. 2(b)]. The PDD curves and gamma values (2%/2 mm) for PDD curves for the 4‐MV, 6‐MV, 6‐MV FFF, and 10‐MV X‐ray beams for the seven square fields with sizes of 3 × 3, 5 × 5, 10 × 10, 15 × 15, 20 × 20, 25 × 25, and 30 × 30 cm^2^ with and without the IQM are shown in Fig. 3. The gamma values (2%/2 mm) for all energies and field sizes from 3 × 3 to 30 × 30 cm^2^ were under 1 except those for the surface of a field size of 30 × 30 cm^2^, where the maximum gamma values were 1.64, 2.17, 2.01, and 2.45 for the 4‐MV, 6‐MV, 6‐MV FFF, and 10‐MV X‐ray beams, respectively. The gamma passing rates (10% threshold) for all energies and field sizes from 3 × 3 to 30 × 30 cm^2^ were above 95% at 2%/2 mm.

### 3.C The effect of IQM on the crossline and inline profiles

**Fig 4:**
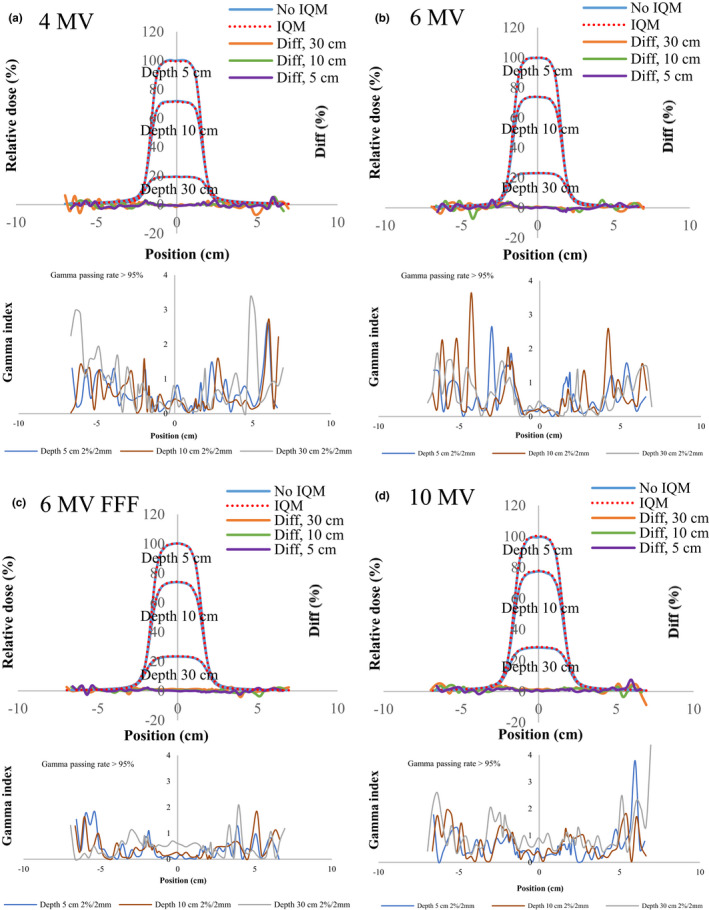
The crossline profiles and gamma values (2%/2 mm) for crossline profiles of (a) 4‐MV, (b) 6‐MV, (c) 6‐MV FFF, and (d) 10‐MV X‐ray beams with and without the IQM at depths of 5, 10, and 30 cm for a field size of 3 × 3 cm^2^ and SSD of 100 cm.

**Fig 5:**
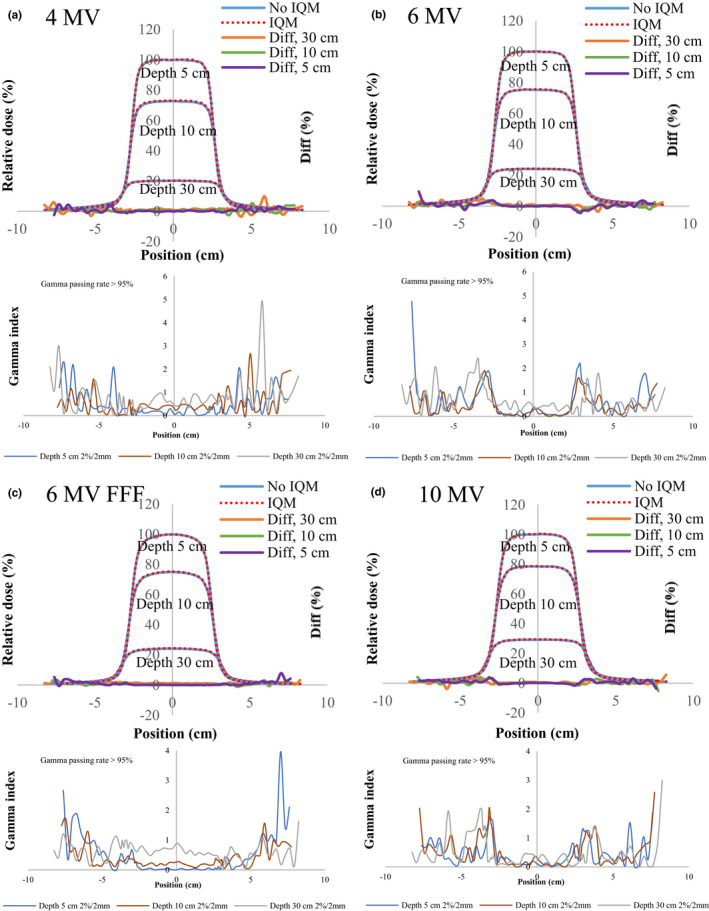
The crossline profiles and gamma values (2%/2 mm) for crossline profiles of (a) 4‐MV, (b) 6‐MV, (c) 6‐MV FFF, and (d) 10‐MV X‐ray beams with and without the IQM at depths of 5, 10, and 30 cm for a field size of 5 × 5 cm^2^ and SSD of 100 cm.

**Fig 6:**
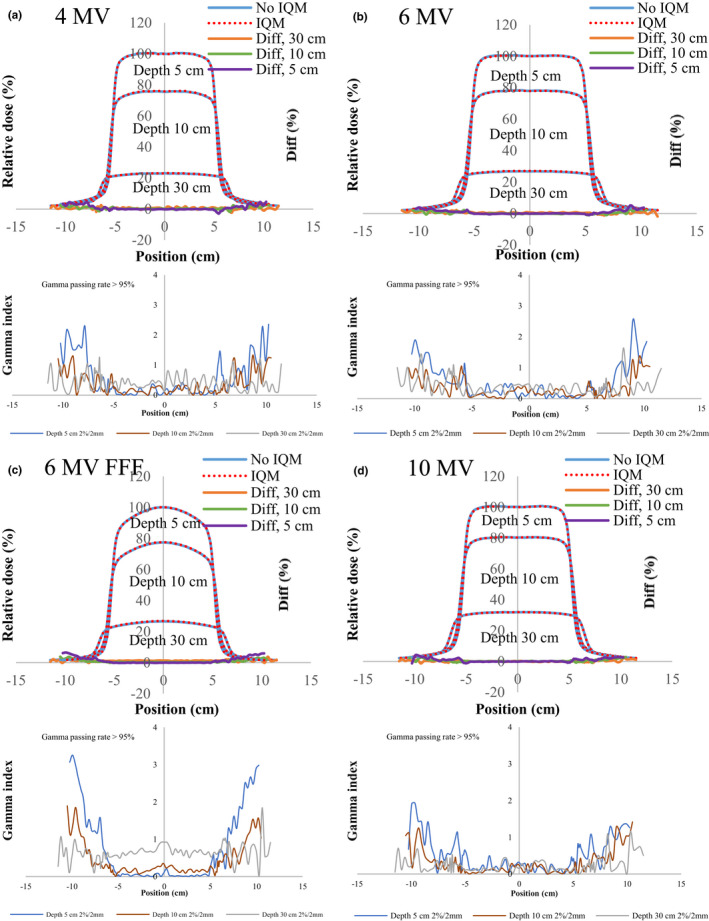
The crossline profiles and gamma values (2%/2 mm) for crossline profiles of (a) 4‐MV, (b) 6‐MV, (c) 6‐MV FFF, and (d) 10‐MV X‐ray beams with and without the IQM at depths of 5, 10, and 30 cm for a field size of 10 × 10 cm^2^ and SSD of 100 cm.

**Fig 7:**
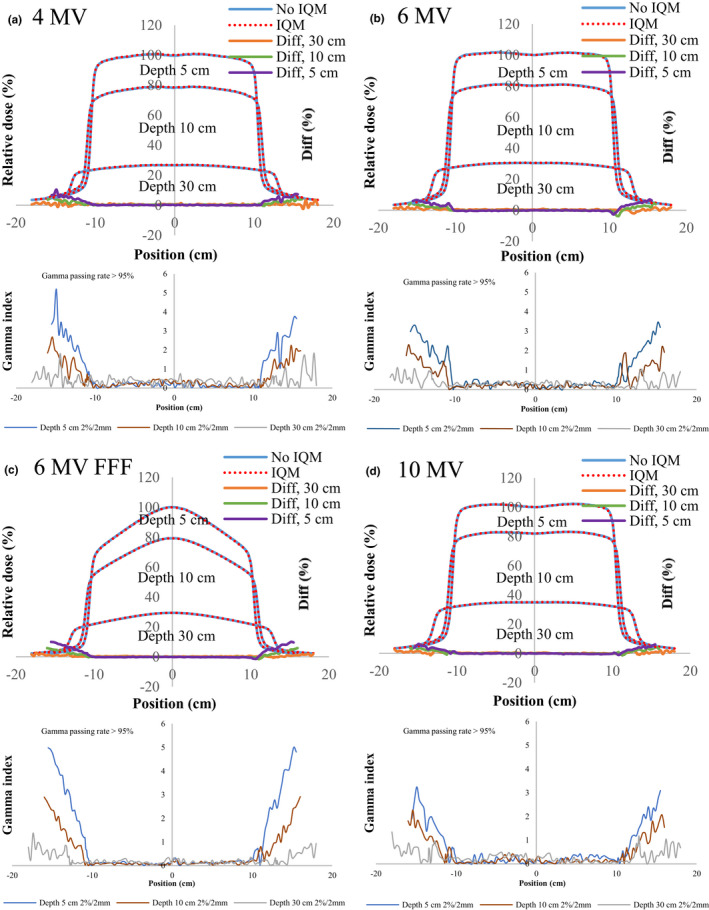
The crossline profiles and gamma values (2%/2 mm) for crossline profiles of (a) 4‐MV, (b) 6‐MV, (c) 6‐MV FFF, and (d) 10‐MV X‐ray beams with and without the IQM at depths of 5, 10, and 30 cm for a field size of 20 × 20 cm^2^ and SSD of 100 cm.

**Fig 8:**
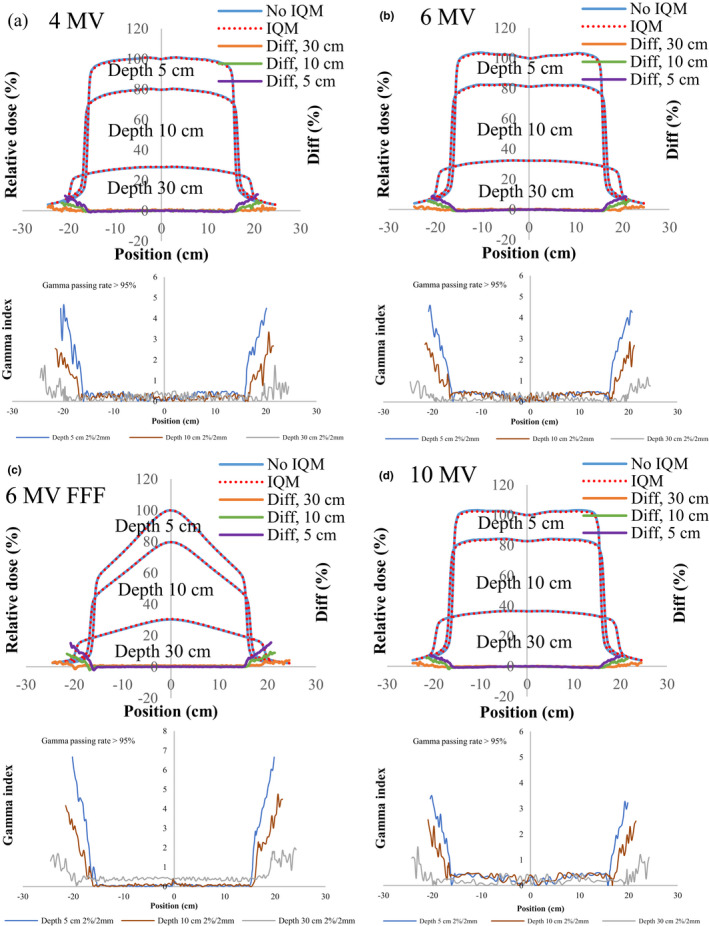
The crossline profiles and gamma values (2%/2 mm) for crossline profiles of (a) 4‐MV, (b) 6‐MV, (c) 6‐MV FFF, and (d) 10‐MV X‐ray beams with and without the IQM at depths of 5, 10, and 30 cm for a field size of 30 × 30 cm^2^ and SSD of 100 cm.

**Fig 9:**
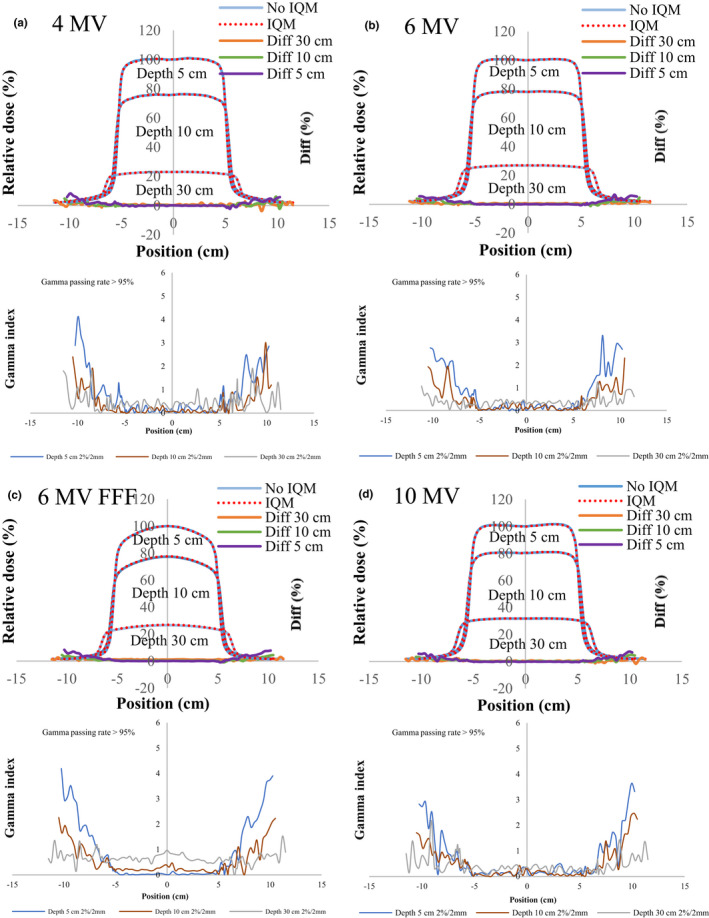
The inline profiles and gamma values (2%/2 mm) for inline profiles of (a) 4‐MV, (b) 6‐MV, (c) 6‐MV FFF, and (d) 10‐MV X‐ray beams with and without the IQM at depths of 5, 10, and 30 cm for a field size of 10 × 10 cm^2^ and SSD of 100 cm.

**Fig 10:**
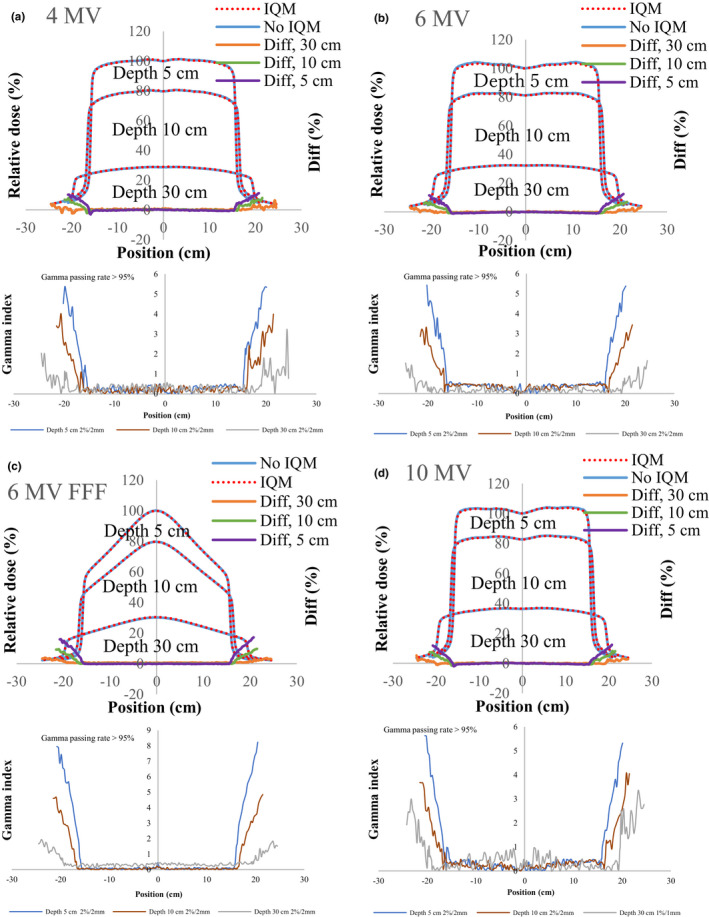
The inline profiles and gamma values (2%/2 mm) for inline profiles of (a) 4‐MV, (b) 6‐MV, (c) 6‐MV FFF, and (d) 10‐MV X‐ray beams with and without the IQM at depths of 5, 10, and 30 cm for a field size of 30 × 30 cm^2^ and SSD of 100 cm.

Figures 4, 5, 6, 7, 8 show the crossline profiles and the corresponding gamma values (2%/2 mm) for crossline profiles of 4‐, 6‐, 6‐MV FFF, and 10‐MV X‐ray beams with and without the IQM at depths of 5, 10, and 30 cm for the five field sizes of 3 × 3, 5 × 5, 10 × 10, 20 × 20, and 30 × 30 cm^2^, respectively. Figures 9 and 10 illustrate the inline profiles and the corresponding gamma values (2%/2 mm) for inline profiles of 4‐, 6‐, 6‐MV FFF, and 10‐MV X‐ray beams with and without the IQM at depths of 5, 10, and 30 cm for the field sizes of 10 × 10 and 30 × 30 cm^2^, respectively. Overall, the crossline and inline profiles measured with the IQM were well matched with the corresponding crossline and inline profiles without the IQM in the central beam region of profiles; however, they were slightly shifted at a depth of 5 cm (within ±1%). At the edge of the dose profiles, the largest differences between the crossline/inline profiles measured with and without the IQM were 15.5% and 17.2% for the crossline and inline profiles, respectively, of the 6‐MV FFF X‐ray beam for the field size of 30 × 30 cm^2^ at a depth of 5 cm [Figs. 8(c) and 10(c)]. The differences at the edge of the crossline profiles for the field size of 30 × 30 cm^2^ ranged from 7.4% (10 MV) to 15.5% (6‐MV FFF) at a depth of 5 cm, 5.9% (10 MV) to 9.5% (6‐MV FFF) at a depth of 10 cm, and 2.4% (6 MV) to 4.2% (6‐MV FFF) at a depth of 30 cm (Fig. 8). The differences at the edge of inline profiles for the field size of 30 × 30 cm^2^ ranged from 11.2% (4 MV) to 17.2% (6‐MV FFF) at a depth of 5 cm, 6.9% (6 MV) to 9.7% (6‐MV FFF) at a depth of 10 cm, and 3.3% (6 MV) to 6.5% (4 MV) at a depth of 30 cm (Fig. 10). By gamma function calculations (2%/2 mm), the crossline/inline profiles measured with IQM showed good agreement with the corresponding crossline/inline profiles measured without IQM in the radiation field regions with gamma values below 1 except those for the edge of crossline/inline profiles. The gamma passing rates (10% threshold) for all energies and field sizes from 3 × 3 to 30 × 30 cm^2^ were above 95% at 2%/2 mm. At the edge of crossline/inline profiles, the gamma values at each energy and each field size decreased with increasing of depth from 5 to 30 cm. The gamma values of each energy at the edge of crossline/inline profiles increased with increase of field sizes from 3 × 3 to 30 × 30 cm^2^ with largest gamma values (2%/2 mm) of 6.67 and 8.23 for the crossline and inline profiles, respectively, of the 6‐MV FFF X‐ray beam for field size of 30 × 30 cm^2^ at a depth of 5 cm [Fig. 8(c) and 10(c)].

### 3.D The effect of IQM on the transmission factors and output factors

**Fig 11:**
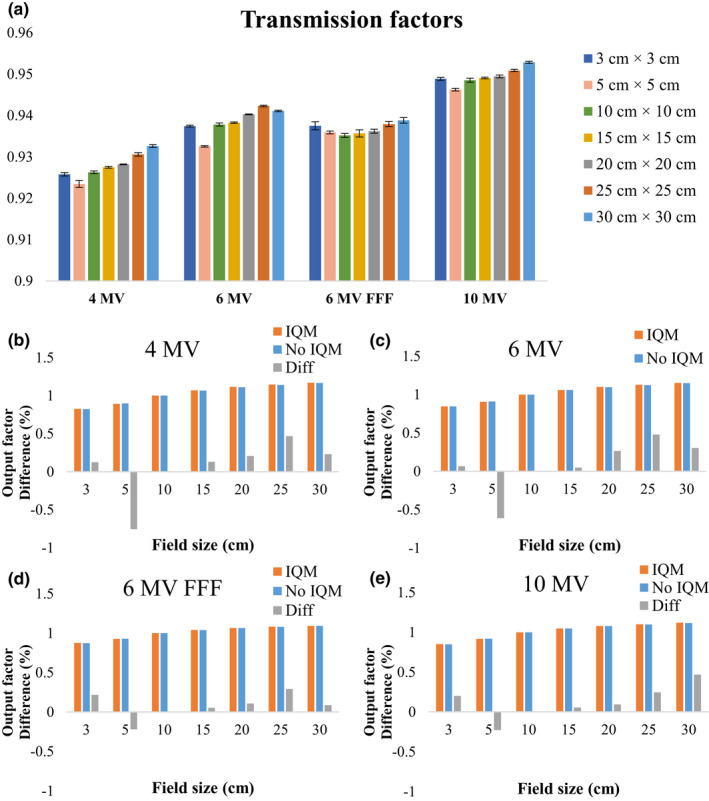
(a) Transmission factors and the differences of output factors for (b) 4‐MV, (c) 6‐MV, (d) 6‐MV FFF, and (e) 10‐MV X‐ray beams at a depth of 10 cm for field sizes of 3 × 3, 5 × 5, 10 × 10, 15 × 15, 20 × 20, 25 × 25, and 30 × 30 cm^2^ and an SCD of 100 cm. The error bars show the standard deviations from five repeated measurements.

The transmission factors for 4‐MV, 6‐MV, 6‐MV‐FFF, and 10‐MV X‐ray beams for field sizes from 3 × 3 to 30 × 30 cm^2^ were 0.926–0.933, 0.937–0.941, 0.937–0.939, and 0.949–0.953, respectively [Fig. 11(a)]. The differences between the lowest and highest transmission factors versus field sizes from 3 × 3 to 30 × 30 cm^2^ for 4‐MV, 6‐MV, 6‐MV FFF, and 10‐MV X‐ray beams were 0.009, 0.009, 0.004, and 0.007, respectively. The differences in output factors of all energies for all field sizes with and without the IQM were in the range from −0.8% (4‐MV) to 0.5% (10‐MV) [Figs. 11(b)–11(e)].

## 4 DISCUSSION

### 4.A The effect of IQM on the surface and build‐up region dose

The results showed that the dose differences at the surface and build‐up region depended on the beam energy and field size. The differences were within 3.7% for field sizes from 3 × 3 to 25 × 25 cm^2^, whereas the discrepancy for a field size of 30 × 30 cm^2^ was up to 8.7%, which was probably because of more collimator scatter when the field size was larger. The presence of the IQM in the beam line increased the dose at the surface and build‐up region when the field size was 30 × 30 cm^2^ because of electron contamination. The secondary electrons from the IQM interacted with the phantom and decreased with increasing depth.^10^, ^12^, ^29^, ^30^ Therefore, the electron contamination in deeper regions was lower compared with that in the surface region.^31^, ^32^ The gamma values (2%/2 mm) were <1 except those for the surface of a field size of 30 × 30 cm^2^, where the maximum gamma values were 1.64, 2.17, 2.01, and 2.45 for the 4‐MV, 6‐MV, 6‐MV FFF, and 10‐MV X‐ray beams, respectively. The difference at the surface dose for the field size of 30 × 30 cm^2^ increased with beam energy from 4 MV (4.6%) to 10 MV (8.7%). Casar et al.^22^ obtained similar results for the difference of surface dose, finding the largest difference of 8.1% for a field size of 20 × 20 cm^2^ (10‐MV FFF X‐ray beam) and within 3.3% for field sizes from 4 × 4 to 15 × 15 cm^2^ (6‐MV, 10‐MV, 15‐MV, 18‐MV, 6‐MV FFF, and 10‐MV FFF X‐ray beam). The magic plate and Dolphin detector showed an increase of surface dose, with a maximum value of 12.1% at the source to detector distance of 80 cm and 11% at SSD of 80 cm for 30 × 30 cm^2^ field size (6‐MV X‐ray beam), respectively.^10^, ^29^ The presence of IBA COMPASS detector in the beam path increased to 8.1% of the surface dose at field size of 20 × 20 cm^2^ and SSD of 90 cm (6‐MV X‐ray beam).^12^

### 4.B The effect of IQM on PDD_20,10_, TPR_20,10_, and PDD curves

We found that there was a small difference in PDD_20,10_ values with and without the IQM for all energies and all field sizes (within 0.6%). Because the differences of PDD at a depth of 20 cm for field sizes of 5 × 5, 10 × 10, 25 × 25, and 30 × 30 cm^2^ of the 6‐MV FFF X‐ray beam were larger than those obtained for the other energies, the differences in PDD_20,10_ of the 6‐MV FFF X‐ray beam for these field sizes were higher than those at the other energies, with the highest value of 0.5% obtained for a field size of 10 × 10 cm^2^. Casar and colleagues showed that the largest difference in PDD_20,10_ for a 6‐MV FFF X‐ray beam was 0.5%.^22^ The differences between TPR_20,10_ with and without the IQM did not exceed 0.3%, with the highest value observed for the 6‐MV FFF X‐ray beam (0.3%) and the lowest value for the 4‐MV X‐ray beam (0.1%). The differences and gamma values (2%/2 mm) in PDD beyond *d*_max_ with and without the IQM for all energies and field sizes were within ±1% and below 1, respectively. The gamma passing rates (10% threshold) for all energies and field sizes from 3 × 3 to 30 × 30 cm^2^ were above 95% at 2%/2 mm. Islam et al.^9^ showed that the differences in PDD in the transient charged particle equilibrium region for a 6‐MV X‐ray beam were within ±1%.

### 4.C The effect of IQM on the crossline and inline profiles

The differences and gamma values (2%/2 mm) in the central region of the dose profiles with and without the IQM for all energies and field sizes were within ±1% and below 1, respectively. The gamma passing rates (10% threshold) for all energies and field sizes from 3 × 3 to 30 × 30 cm^2^ were above 95% at 2%/2 mm. Islam et al.^9^ showed that the dose profiles at the central region for a 6‐MV X‐ray beam were within ±1%. However, the differences at the edge of the dose profiles measured with and without the IQM in this study were up to 17.2% for the inline profile (6‐MV FFF) and 15.5% for the crossline profile (6‐MV FFF) for field size of 30 × 30 cm^2^ at a depth of 5 cm. The gamma values at the edge of the dose profiles were up to 4.4 and 5.5 for the crossline and inline profiles, respectively, of the 6‐MV FFF X‐ray beam for field size of 30 × 30 cm^2^ at a depth of 5 cm.

### 4.D The effect of IQM on the transmission factors and output factors

When the IQM was placed in the beam path, the beam attenuation of the IQM decreased with increasing X‐ray beam energy from 4 to 10 MV and decreased with increasing field size from 3 × 3 to 30 × 30 cm^2^. The transmission factors of square fields with sizes from 3 × 3 to 30 × 30 cm for 6‐MV, 10‐MV, and 6‐MV FFF X‐ray beam were 0.94, 0.95, and 0.94, respectively. Casar et al.^22^ found that mean transmission factors of all square fields from 1 × 1 to 20 × 20 cm^2^ for 6‐MV, 10‐MV, and 6‐MV FFF multicenter X‐ray beams were 0.94, 0.95, and 0.94, respectively. This study showed that the IQM attenuated the 6‐MV beam with a field size of 10 × 10 cm^2^ by 6.2%. Islam and co‐workers revealed that the IQM attenuated a 6‐MV beam with a field size of 10 × 10 cm^2^ by 7%.^9^

The differences of output factors varied as a function of the field size and were within ±1% with the lowest values observed for the 6‐MV X‐ray beam (0.1% for 3 × 3 cm^2^) and 6‐MV FFF X‐ray beam (−0.2%, 0.1%, 0.1%, 0.3%, and 0.1% for 5 × 5, 5 × 15, 20 × 20, 25 × 25, and 30 × 30 cm^2^, respectively). The backscatter from the IQM may affect the differences in output factors with and without the IQM. For a field size of 5 × 5 cm^2^, the output factors measured with the IQM for all energies were lower than those measured without the IQM. In contrast, for field sizes of 3 × 3, 15 × 15, 20 × 20, 25 × 25, and 30 × 30 cm^2^, the output factors measured with the IQM for all energies were higher than those measured without the IQM.

The difference in the surface dose when the field size was 30 × 30 cm^2^ for the 6‐MV FFF X‐ray beam was smaller than those for the 6‐ and 10‐MV X‐ray beam because the FFF conditions decreased the electrons contamination.^30^ The differences in PDD_20,10_, TPR_20,10_, and crossline/inline profiles of the 6‐MV FFF X‐ray beam with and without the IQM were larger than those of the conventional flattening filter photon beams (4‐, 6‐, and 10‐MV X‐ray beams), whereas the differences in output factors were smaller compared with those obtained for beams with other energies. The flattening filter eliminated the primary photons. Therefore, the edge of the field of the FFF beam received a higher head scatter dose compared with that for the edge of beams with the flattening filter.

The differences in surface and build‐up dose, PDD_20,10_, and TPR_20,10_ of the 4‐MV X‐ray beam with and without the IQM were smaller than those obtained at other energies. However, the beam attenuation of the IQM for the 4‐MV X‐ray beam was higher than that of beams with other energies. Therefore, a tray factor should be put into treatment planning systems for dose calculation for the 4‐MV X‐ray beam. Casar et al.^22^ suggested to configure treatment planning system through tray factors or modify output factors for particular beam energy before using IQM in pre‐ and intra‐ treatment QA. Therefore, it is necessary to evaluate the commissioning of the IQM device whether the changes in the beam characteristics and output factors could account for the attenuation of IQM. Some papers showed that the IQM system has the potential in its clinical use. Marrazzo et al.^33^ reported the IQM detector is a highly sensitive dose‐monitoring device for clinical practice of step‐and‐shoot IMRT plan and found a good correlation between the measured IQM signal and DVH metrics that is useful for identifying clinical action levels. Esposito et al.^20^ performed the IQM that is a useful in vivo dosimetry tool with the strengths of real‐time monitoring of linac status and can monitor all fractions. The differences in surface and build‐up dose, PDD_20,10_, PDD curves, and crossline/inline profiles, and attenuation of the IQM for 6‐ and 10‐MV X‐ray beams with and without the IQM were relatively similar to the findings published in previous papers.^9^, ^22^, ^23^

The differences of crossline/inline profiles with and without the IQM increased from the end position of the field width to the end of the crossline/inline profiles, which might be related to reasons such as the radiation component of secondary electrons, beam hardening effect, and backscattered radiation from the aluminum plates (alloy 6061) of the IQM. Moreover, the Infinity Agility MLC system has a pair of backup jaws that are orthogonal to the direction of leaf motion. Therefore, the scatter from sculpted field‐length defining collimators may affect the edge of the inline profile. The differences and gamma values at the outside region of the fields of crossline/inline profiles with and without the IQM decreased with increasing depth in the order of 5 to 10 to 30 cm, which was probably caused by the presence of contaminating electrons from the treatment head and IQM at shallow depth.^29^ As a limitation of this study, the cause of the differences at the toe of crossline/inline profiles with and without the IQM is still unclear. Therefore, it is necessary to evaluate photon energy properties with and without the IQM, factors influencing beam characteristics, and backscatter contribution using Monte Carlo simulations and to verify the simulation results with the measured data. The differences at the toe of crossline/inline profiles measured with and without the IQM showed whether the presence of the IQM affects MLC‐generated photon fluence in IMRT technique dosimetry. Therefore, the ability of using the IQM detector in verification of IMRT delivery should be investigated.

## 5 CONCLUSION

We evaluated the difference in the beam quality measured with and without an IQM and found the field‐size and beam‐energy dependence of the IQM. The influence of IQM on the beam quality (in particular 4‐MV X‐ray has not verified before) was tested and introduced a slight beam perturbation at the surface and build‐up region and the edge of the crossline/inline profiles. To use IQM in pre‐ and intra‐treatment QA, a tray factor should be put into treatment planning systems for the dose calculation for the 4‐, 6‐, 10‐MV, and 6‐MV flattening filter‐free photon beams to compensate for the attenuation of the IQM detector.

## Conflict of Interest

This study was a collaborative research project with APEX Medical, Inc."

## AUTHOR CONTRIBUTION STATEMENT

Trang Hong Thi Nguyen, Conceived and designed the analysis, collected the data, contributed data or analysis tools, performed the analysis, wrote the paper, and revised the paper. Haruna Yokoyama; Collected the data, contributed data or analysis tools, performed the analysis, reviewed and revised the paper. Hironori Kojima, Collected the data, contributed data or analysis tools, performed the analysis, reviewed and revised the paper. Naoki Isomura, Collected the data, contributed data or analysis tools, performed the analysis, reviewed and revised the paper. Akihiro Takemura, Collected the data, contributed data or analysis tools, performed the analysis, reviewed and revised the paper. Shinichi Ueda, Collected the data, contributed data or analysis tools, performed the analysis, reviewed and revised the paper. Kimiya Noto, Collected the data, contributed data or analysis tools, performed the analysis, reviewed and revised the paper.
